# Routes of Clonal Evolution into Complex Karyotypes in Myelodysplastic Syndrome Patients with 5q Deletion

**DOI:** 10.3390/ijms19103269

**Published:** 2018-10-21

**Authors:** Simone Feurstein, Kathrin Thomay, Winfried Hofmann, Guntram Buesche, Hans Kreipe, Felicitas Thol, Michael Heuser, Arnold Ganser, Brigitte Schlegelberger, Gudrun Göhring

**Affiliations:** 1 Department of Human Genetics, Hannover Medical School, Carl-Neuberg-Str.1, 30625 Hannover, Germany; feurstein@uchicago.edu (S.F.); Thomay.Kathrin@mh-hannover.de (K.T.); Hofmann.Winfried@mh-hannover.de (W.H.); Schlegelberger.Brigitte@MH-Hannover.de (B.S.); 2 Department of Medicine, Section of Hematology/Oncology, The University of Chicago, 900 E 57th street, Chicago, IL 60637, USA; 3 Institute of Pathology, Hannover Medical School, Carl-Neuberg-Str.1, 30625 Hannover, Germany; Buesche.Guntram@mh-hannover.de (G.B.); Kreipe.Hans@mh-hannover.de (H.K.); 4 Department of Hematology, Hemostasis, Oncology, and Stem Cell Transplantation, Hannover Medical School, Carl-Neuberg-Str.1, 30625 Hannover, Germany; Thol.Felicitas@mh-hannover.de (F.T.); Heuser.Michael@mh-hannover.de (M.H.); Ganser.Arnold@mh-hannover.de (A.G.)

**Keywords:** myelodysplastic syndrome, clonal evolution, chromothripsis, complex karyotype, *TP53*

## Abstract

Myelodysplastic syndrome (MDS) can easily transform into acute myeloid leukemia (AML), a process which is often associated with clonal evolution and development of complex karyotypes. Deletion of 5q (del(5q)) is the most frequent aberration in complex karyotypes. This prompted us to analyze clonal evolution in MDS patients with del(5q). There were 1684 patients with low and intermediate-risk MDS and del(5q) with or without one additional cytogenetic abnormality, who were investigated cytogenetically in our department, involving standard karyotyping, fluorescence in situ hybridization (FISH) and multicolor FISH. We identified 134 patients (8%) with aspects of clonal evolution. There are two main routes of cytogenetic clonal evolution: a stepwise accumulation of cytogenetic events over time and a catastrophic event, which we defined as the occurrence of two or more aberrations present at the same time, leading to a sudden development of highly complex clones. Of the 134 patients, 61% underwent a stepwise accumulation of events whereas 39% displayed a catastrophic event. Patients with isolated del(5q) showed significantly more often a stepwise accumulation of events rather than a catastrophic event. The most frequent aberrations in the group of stepwise accumulation were trisomy 8 and trisomy 21 which were significantly more frequent in this group compared to the catastrophic event group. In the group with catastrophic events, del(7q)/-7 and del(17p)/-17 were the most common aberrations. A loss of 17p, containing the tumor suppressor gene *TP53*, was found significantly more frequent in this group compared to the group of stepwise accumulation. This leads to the assumption that the loss of *TP53* is the driving force in patients with del(5q) who undergo a sudden catastrophic event and evolve into complex karyotypes.

## 1. Introduction

Myelodysplastic syndromes (MDS) are a heterogeneous group of clonal hematopoietic stem cell disorders characterized by ineffective and dysplastic hematopoiesis and increased risk of transformation into acute myeloid leukemia (AML) [1,2]. Chromosomal abnormalities can be detected in approximately 50% of patients with de novo MDS and in up to 80% of patients with therapy-related MDS [3,4]. A complex karyotype, defined as at least three clonal aberrations, is detected in approximately 10% to 15% of MDS patients, and is associated with a very short median survival of less than 12 months and a high risk of transformation into AML [3,4,5,6].

MDS with isolated deletion of 5q (del(5q)) is a unique entity, which has been expanded by the revised World Health Organization (WHO) classification in 2016 and now includes patients with del(5q) and one additional cytogenetic abnormality with the exception of monosomy 7 or del(7q). The outcome of patients with del(5q) with or without an additional cytogenetic aberration depends on the presence of clonal evolution, cytopenia, and excess of blasts in the bone marrow, which subsequently worsens the outcome and increases the risk of transformation into AML [1,7,8].

Among others, we have recently described that leukemic progression in low and intermediate-risk MDS with isolated del(5q) is associated with clonal evolution and identified *TP53* mutations and excessive telomere shortening as driving forces for clonal evolution and leukemic progression [9,10,11,12,13]. Clonal evolution determines the clinical course in myeloid malignancies based on the interaction of selectively advantageous “driver” lesions, selectively neutral “passenger” lesions and harmful lesions [14,15]. In MDS, the modes of clonal evolution and the impact of potential “driver” lesions leading to disease progression and transformation to AML have remained largely unclear and mechanisms responsible for the induction of chromosomal instability and the development of complex karyotypes are still poorly understood. In general, there appear to be two different routes of clonal evolution. One is characterized by stepwise acquisition of additional aberrations resulting in clonal selection of clones that had accumulated mostly only one or two additional aberrations according to the so-called “Vogelstein model” [16]. In contrast, recent studies identified a process called “chromothripis”, a sudden catastrophic event which leads to massive chromosomal rearrangements and shattering of entire chromosomes [17,18,19]. Although MDS with del(5q) is assumed to be a relatively genetically stable hematologic neoplasm, clonal evolution, even into complex karyotypes, occurs in a significant proportion of patients, which prompted us to further investigate clonal evolution in this subgroup. 

In this work, we investigated a large cohort of 1684 patients with del(5q), with or without one additional aberration, who have received cytogenetic testing at our institute. In the evaluation process, we distinguished between two different routes of clonal evolution, the “stepwise accumulation” of aberrations and a “catastrophic event” similar to chromothripsis, and analyzed the frequencies of specific aberrations in both cohorts.

## 2. Results

We identified 1684 patients with low- and intermediate-risk MDS and del(5q) whom were investigated cytogenetically at our institute using standard karyotyping and fluorescence in situ hybridization (FISH) analyses. Of those, 161 out of the 1684 patients showed additional cytogenetic aberrations which were either present at the time of diagnosis or developed over time during multiple cytogenetic analyses. We were able to show that 134 out of the 161 patients developed additional aberrations within the del(5q) clone defined as clonal evolution. This accounted for 8% of the initial cohort of 1684 patients. The other 27 out of the 161 patients showed independent clones not present within the del(5q) clone.

For 94 out of the 161 patients, cytogenetic follow-up data were available (follow-up from 0–66 months, median 16 months). In the 67 patients without, independent clones or additional chromosome aberrations in subclones due to clonal evolution were detected at the time point of diagnosis. For the 94 patients with available follow-up data, clonal evolution was identified whenever the cytogenetic term “idem” was used to describe a subclone with del(5q) and additional aberrations or when a patient with a primary isolated del(5q) clone showed additional aberrations within this clone during follow-up. In order to categorize the 67 patients without follow-up data we again used the cytogenetic term “idem” and the number of additional aberrations in subclones at the time point of diagnosis. We further subdefined the group of patients with clonal evolution (*n* = 134) into two groups of patients, the first group of patients which initially harbored an isolated del(5q) as the sole cytogenetic abnormality (*n* = 112/134, 84%) and a second group of patients with del(5q) and one additional aberration (*n* = 22/134, 16%) present at the first time of cytogenetic testing. 

These two groups of patients undergoing clonal evolution were further subcategorized into two evolution modes, a stepwise accumulation of additional cytogenetic events (*n* = 82/134, 61%) and a catastrophic event (*n* = 52/134, 39%). All groups and subgroups including examples of karyotypes are shown in Table 1.

**Figure 1:**
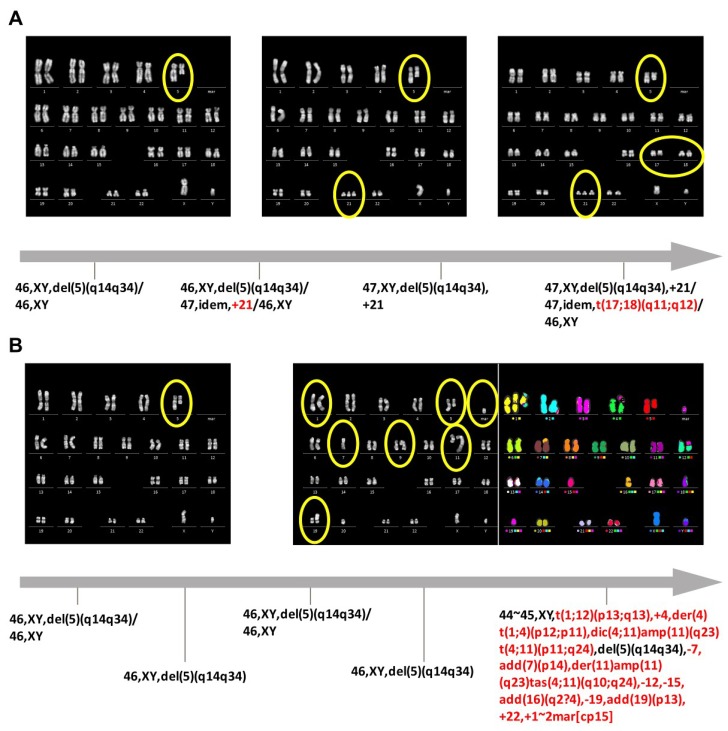
Depicted are examples of cytogenetic results (karyograms after fluorescence R-banding and after multicolor fluorescence in situ hybridization (mFISH)), which show the two different routes of clonal evolution. Additional aberrations gained over time are highlighted in red in the karyotype and with yellow circles within the karyograms. (**A**) Shows a patient with stepwise acquisition of additional aberrations during follow-up. The patient first gained an additional chromosome 21, and over time another translocation t(17;18), which then presented as a complex karyotype. (**B**) Shows the sudden development of a highly complex clone, which we defined as clonal evolution due to a catastrophic event.

We defined stepwise accumulation according to the Vogelstein model as the acquisition of one additional aberration at the time, which may or may not lead to a complex karyotype (conventionally defined as three or more chromosome abnormalities) over time. An example for this group is a patient with the karyotype 46,XX,del(5)(q14q34)[10]/47,idem,+8[5]. Additional examples are shown in Table 1. The karyogram of a patient with stepwise accumulation can also be seen in Figure 1A. A stepwise accumulation was the underlying pathway of clonal evolution in 70 out of 112 patients with initial isolated del(5q) clone (63%) and in 12 out of 22 patients with one additional aberration present within the del(5q) clone (54%). The number of gained aberrations ranged from 1 to 8 (median 1).

A catastrophic event was classified as acquisition of two or more cytogenetic aberrations within the del(5q) clone at the same time of cytogenetic analysis, automatically resulting in a complex karyotype in these patients. As an example of this group, the patient could harbor the following karyotype: 46,XX,del(5)(q14q34)[10]/47,idem,+8,del(12)(p12p13)[5], additional examples are shown in Table 1. The karyogram and multicolor (m)FISH results of a patient with a sudden catastrophic event with gain of a multitude of aberrations is presented in Figure 1B. A catastrophic event was the underlying mechanism in 42 out of 112 patients with initial isolated del(5q) clone (38%), and 10 out of 22 patients with one additional aberration present within the del(5q) clone (46%). The number of gained aberrations in this subgroup frequently resulted in highly complex clones, ranging from 2 to 27 (median 5).

In MDS with isolated del(5q), a stepwise accumulation occurred significantly more often than a catastrophic event (*p* = 0.04). There was no significant difference between the frequency of either route in patients with del(5q) and one additional aberration. The median age at diagnosis for the group with stepwise accumulation was 68.7, and for the group with catastrophic event, 66.1. There was no significant difference in the age at diagnosis between the two groups (*p* = 0.96). Additional clinical data were not available.

Complex karyotypes developed in 76 out of 134 patients (57%) undergoing clonal evolution. As cytogenetically defined, all patients with a catastrophic event (*n* = 52) developed a complex karyotype. The 22 patients with one additional aberration within the del(5q) clone evolved into complex karyotypes either by stepwise accumulation (*n* = 12) or a catastrophic event (*n* = 10). Out of the 112 patients with isolated del(5q), 12 were found to display a complex karyotype due to stepwise accumulation and 42 due to a catastrophic event.

The time from initial diagnosis of MDS with del(5q) to stepwise progression (irrespective of the development of a complex karyotype) ranged from 1 to 51 months (median 7 months) whereas a catastrophic event appeared on average after 12 months (range 1 to 62 months). The development of a complex karyotype occurred at a median of 12 months in both the stepwise progression and catastrophic event groups. 

**Figure 2:**
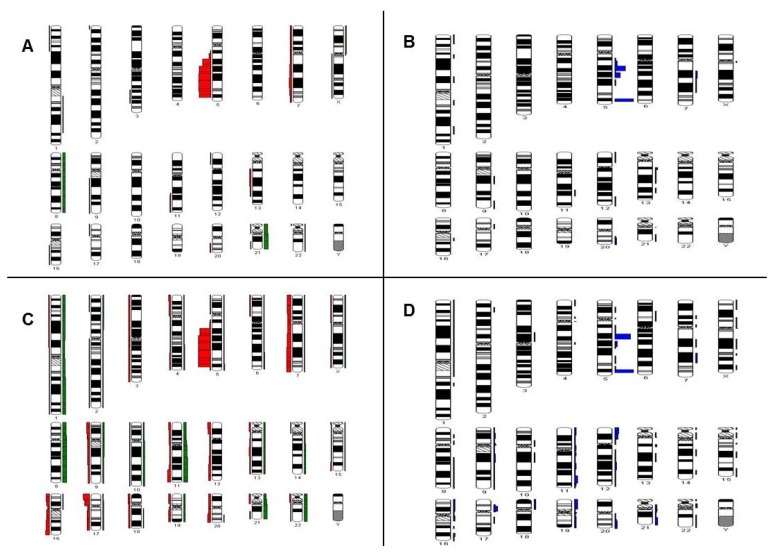

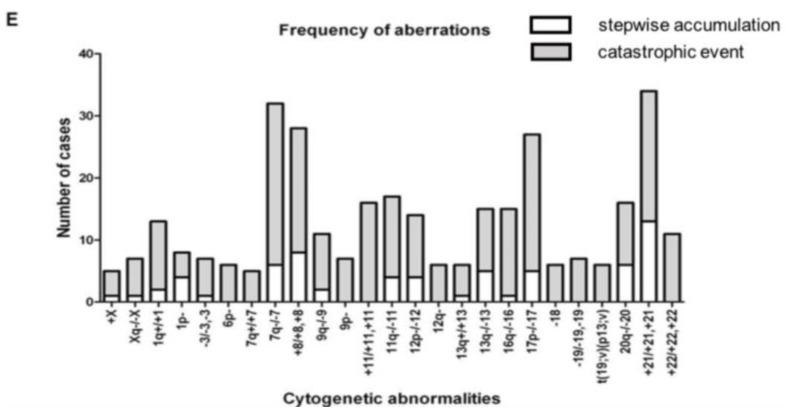
(**A**–**D**) Results of the CyDAS evaluation of patients with a stepwise accumulation of aberrations (**A**,**B**) or catastrophic events (**C**,**D**). Gains are shown as green bars on the right side of a chromosome, losses are shown as red bars on the left side of a chromosome. A greater thickness of the bars represents a higher frequency of these aberrations in the groups analyzed and vice versa. Recurrent breakpoints are shown in blue on the right side of the chromosome. A greater thickness of the bars represents a higher frequency of these breakpoints in the groups analyzed and vice versa. (**A**) Numeric changes in patients with a stepwise accumulation of events. (**B**) Chromosomal breakpoints in patients with a stepwise accumulation of events. (**C**) Numeric changes in patients with a catastrophic event. (**D**) Chromosomal breakpoints in patients with a catastrophic event. (**E**) Frequency of the 25 most common cytogenetic aberrations subdivided into occurrence during stepwise accumulation or as a catastrophic event leading to complex karyotypes.

We evaluated the karyotypic data from R-banding and FISH with the Cytogenetic Data Analysis System (CyDAS) [20] for recurrent gains/losses and recurrent breakpoints (Figure 2A–D), which allowed us to visualize common aberrations in both groups. We detected significant differences in the occurrence of certain aberrations in each route of clonal evolution.

The most frequent additional aberration gained over time as stepwise accumulation in both the group of patients with initial isolated del(5q) clone or del(5q) with one additional aberration, was a gain of chromosome 21 (Figure 2A,E). Other frequent abnormalities found in this group (in order of decreasing frequency) were gain of chromosome 8, del(7q)/-7, del(20q)/-20, del(13q)/-13, del(17p)/-17, del(1p), del(11q)/-11 and del(12p)/-12 (Figure 2A,E). Trisomy 8 and 21 occurred at a significantly higher frequency in the process of stepwise acquisition than as part of catastrophic events (*p* = 0.008 and *p* = 0.00001, respectively).

Frequent breakpoints shown in Figure 2B are located within the long arms of chromosomes 7, 13, and 20.

In the context of catastrophic events leading to complex karyotypes, the most frequent abnormalities found were del(17p)/-17 and del(7q)/-7 in 40% and 38% of the patients, respectively (Figure 2C,E). Other frequent abnormalities found in this group (in order of decreasing frequency) were trisomy/tetrasomy 21, trisomy/tetrasomy 8, trisomy/tetrasomy 11, del(16q)/-16, del(11q)/-11, gain of chromosome 1 or the long arm of chromosome 1, trisomy/tetrasomy 22, del(13q)/-13, del(20q)/-20 and del(12p)/-12 (Figure 2C,E). Aberrations only present in complex karyotypes were del(6p), gain of chromosome 7 or the long arm of chromosome 7, del(9p), trisomy or tetrasomy 11, del(12q), monosomy 18, monosomy 19 or nullisomy 19, translocation involving the short arm of chromosome 19 (19p13) with various translocation partners, and trisomy or tetrasomy 22 (Figure 2E). A deletion of 17p13 (*TP53*) occurred significantly more often in the catastrophic events group than in the stepwise accumulation group (*p* < 0.0001). Frequent breakpoints in this group involved the long arms of chromosomes 7, 20 and 21, the short arm of chromosome 17 and throughout chromosomes 11, 12, 16, and 19 (Figure 2D).

## 3. Discussion

Clonal evolution is not frequent in patients with MDS and del(5q) but if it occurs, it often results in disease progression and the emergence of a complex karyotype [3,9]. Accordingly, in this study, we were able to demonstrate that 8% of the patients in our large cohort of 1684 MDS patients with del(5q) with or without one additional aberration, underwent clonal evolution.

We identified two main routes of clonal evolution- a stepwise acquisition of aberrations and a catastrophic event leading to the development of complex karyotypes. Stepwise accumulation was the main pathway in patients with isolated del(5q) (70 out of 112) and occurred significantly more frequently in this group than a catastrophic event (*p* = 0.04). In patients with one additional aberration, both routes seemed to occur at the same frequency. A catastrophic event with sudden occurrence of at least two cytogenetic events was identified in 42 patients with isolated del(5q) and 10 patients with del(5q) and one additional aberration. Patients with del(5q) and one additional cytogenetic aberration appeared to be more prone to suffer a catastrophic event that leads to a complex karyotype. This may be due to the second cytogenetic aberration, which confers a higher chromosomal instability and greater likelihood of gaining further aberrations. In 2016, the revised WHO classification expanded the entity of del(5q) to either isolated del(5q) or del(5q) with one additional aberration [1]. So far, no difference in the survival between the groups of single versus double cytogenetic abnormalities has been found [8,21].

We analyzed recurrent aberrations in both groups and found certain specific aberrations to be more common in one group or the other. The stepwise accumulation often involved the gain of chromosomes 21 (the most frequent aberration in this group) and 8, both occurred at a significantly higher frequency compared to their involvement in complex karyotypes (*p* = 0.00001 and *p* = 0.008, respectively). Both of these aberrations, if occurring as single abnormalities, correspond to an intermediate cytogenetic risk score according to the Revised International Prognostic Scoring System [2]. This may explain why these aberrations are more frequent in the group of stepwise accumulation of cytogenetic events that leads less often to complex karyotypes. 

Conversely, in the group of patients who suffered a catastrophic event resulting in a complex karyotype, we found del(17p)/−17 and del(7q)/−7 as the most frequent abnormalities. The deletion of 17p13, involving the tumor suppressor gene *TP53*, occurred significantly more often in the catastrophic events group than in the stepwise accumulation group (*p* < 0.0001). We also detected the loss of 17p, harboring the *TP53* gene, being a recurrent aberration as the “second hit” in our cohort with del(5q) which, during further follow-up, suffered a catastrophic event.

Since we did not have cytogenetic follow-up data on all patients, and the time between the cytogenetic follow-ups varied from patient to patient depending on the clinical need for another bone marrow biopsy, we cannot rule out the possibility that some of the seemingly catastrophic events may actually have been a more sequential but rapid process of accumulating aberrations. Moreover, even with a thorough analysis with R-banding and FISH, small clones may not have been detected at the time of testing.

However, there is evidence, that *TP53* mutations are associated with chromothripsis [22], and telomere shortening and *TP53* mutations are driving forces for clonal evolution and leukemic progression [6,9,11,23]. This supports our findings of del(17p) occurring significantly more often in patients with a catastrophic event leading to highly complex clones or as the sole abnormality preceding such an event. 

There are cytogenetic aberrations that were only found as part of complex karyotypes in this study (see Figure 2E). A possible cause may be greater chromosomal instability leading to rather infrequent cytogenetic aberrations in MDS. Some of these aberrations may represent passenger aberrations that do not necessarily promote disease progression.

In summary, we determined that the frequency of clonal evolution in patients with del(5q) is 8%. Of the patients with isolated del(5q), patients underwent clonal evolution significantly more often by stepwise accumulation than as a catastrophic event. Patients with del(5q) and one additional aberration did not show a significant difference in regards to the route of clonal evolution. Both trisomy 8 and 21 were significantly more frequent in the group of stepwise accumulation, while patients with catastrophic events showed a high rate of del(7q)/−7 or del(17p)/−17. The deletion of the short arm of chromosome 17, resulting in the loss of *TP53*, occurred significantly more often in patients with a single catastrophic event and may represent the driver lesion for clonal evolution, disease progression and leukemic transformation in patients with del(5q).

## 4. Material and Methods

### 4.1. Patient Characteristics

We analyzed data of 1684 patients with low and intermediate-risk MDS and del(5q) investigated cytogenetically in our institute. Out of the 1684 patients, there were 134 carrying either an isolated del(5q) or a del(5q) with one additional aberration at the first time of testing, who underwent clonal evolution. There were 27 patients found to carry independent cytogenetic clones and were not further analyzed. There were 119 patients who were female and 42 patients who were male, with a female to male ratio of 3:1. The diagnosis of MDS was made at Hannover Medical School according to the most recent WHO criteria at the time. Written informed consent was obtained according to the Declaration of Helsinki and the study was approved by the Human Research Ethics Committee at Hannover Medical School (ID 2899, 26/02/2002).

### 4.2. Fluorescence R-Banding and Analysis of Karyotypes

Cytogenetic analyses were performed on short-term cultures (24–48 h) of bone marrow aspirate. Cell cultivation, chromosome preparation and staining for Fluorescence R-banding were performed as described in detail earlier [24]. Whenever possible, we examined 25 metaphases per patient. The karyotype, including all numerical and structural changes, was described according to guidelines of the International System for Human Cytogenetic Nomenclature (ISCN 2016).

### 4.3. FISH

FISH on interphase nuclei was performed as described earlier [25]. If enough material was available, at least 200 interphase nuclei were analyzed for every probe, e.g., EGR1 (5q31), TP53 (17p13), CEP8 and CEP7/7q31 (Abbott, Wiesbaden, Germany). Cut-off levels for positivity were established by analyzing at least 1000 cells from 10 healthy donors and ranged from 5% to 10%.

### 4.4. mFISH

mFISH analysis was carried out using a human chromosome-specific mFISH kit (MetaSystems, Altlussheim, Germany). The mFISH procedure was performed according to the manufacturers’ instructions and as previously described [26]. Fluorochromes were sequentially captured using specific single-band pass filters in a Zeiss Axioplan 2 microscope (Zeiss, Jena, Germany). mFISH ISIS software (MetaSystems, Altlussheim, Germany) was used for image analysis. At least five metaphases were analyzed.

### 4.5. Statistical Analysis

We used the two-tailed Fisher’s exact test, the chi-squared test and the log-rank test. An effect was considered significant if the two-sided *p*-value was <0.05.

## Figures and Tables

**Table 1:** Overview of our patient cohort, the two subgroups of clonal evolution (stepwise accumulation and catastrophic event) and examples of karyotypes for each subgroup and route of clonal evolution.

**Total Cohort**		
Patients with del(5q)		1684
Patients with acquired additional aberrations at the time of diagnosis/during follow-up		161
Patients with clonal evolution within the del(5q) clone		134
Patients with independent clones		27
**Subcategories**		
**Isolated Del(5q)**	**Number**	**Example**
Initial cytogenetic status at first time point	112/134	46,XX,del(5)(q14q34)[14]/46,XX[6]
Stepwise accumulation of cytogenetic events	70	
*-*Not resulting in complex karyotypes	58	46,XX,del(5)(q14q34)[3]/47,idem,+21[17]
*-*Resulting in complex karyotypes	12	46,XX,del(5)(q14q34)[3]/47,idem,+21[10]/47,idem,del(12)(p12p13),+21[7]
Catastrophic event *	42	46,XX,del(5)(q14q34)[3]/44,idem,-7,dic(8;17)(p11;p11),del(12)(p12p13),-18[17]
**Del(5q) and One Additional Aberration**	**Number**	**Example**
Initial cytogenetic status at first time point	22/134	46,XX,del(5)(q14q34),del(20)(q12q13)[11]/46,XX[9]
Stepwise accumulation of cytogenetic events	12	
*-*Not resulting in complex karyotypes	0	
*-*Resulting in complex karyotypes	12	46,XX,del(5)(q14q34),del(20)(q12q13)[4]/46,idem,del(11)(q14) [16]
Catastrophic event *	10	46,XX,del(5)(q14q34),del(20)(q12q13)[5]/46,idem,t(4;16)(q32;q12),del(7)(q21q31),+8,-17,add(22)(q12) [15]

* defined as acquisition of two or more cytogenetic changes at the same time, automatically resulting in a complex karyotype. Deletion of 5q: del(5q).

## Abbreviations

AML	Acute myeloid leukemia
del(5q)	Deletion of 5q
CyDAS	Cytogenetic Data Analysis System
FISH	Fluorescence in situ hybridization
MDS	Myelodysplastic syndrome
m	Multicolor
WHO	World Health Organization
